# A Framework for Effective Bt Maize IRM Programs: Incorporation of Lessons Learned From *Busseola fusca* Resistance Development

**DOI:** 10.3389/fbioe.2020.00717

**Published:** 2020-07-10

**Authors:** Gustav Bouwer

**Affiliations:** Invertebrate Pathology and Biocontrol Laboratory, School of Molecular and Cell Biology, University of the Witwatersrand, Johannesburg, South Africa

**Keywords:** insect resistance management, *Bacillus thuringiensis*, Cry1Ab, MON810, *Busseola fusca*, refuge compliance

## Abstract

Bt maize is genetically engineered to express insecticidal proteins from the bacterium *Bacillus thuringiensis*. Bt maize is used extensively by South African farmers to reduce yield losses caused by lepidopteran larvae. Starting in the 2004/2005 season, severe *Busseola fusca*-associated damage to Cry1Ab-expressing Bt maize was noted by South African farmers. The unsatisfactory pest control was eventually attributed to the development of insect resistance to the Cry1Ab protein in the Bt maize hybrids. An assessment of the historical events surrounding the development of resistance by *B. fusca* showed that there was room for improvement both in the insect resistance management (IRM) strategy selected and the implementation of the strategy. With the recent arrival of fall armyworm (*Spodoptera frugiperda*) in Africa, it is important to have IRM programs that are appropriate for all of the pests that constitute the maize lepidopteran pest complex. After the identification of shortcomings in the IRM programs implemented in South Africa, a framework is proposed for effective Bt maize IRM programs. The IRM framework integrates pre-marketing research, post-marketing monitoring, and two-level remedial action plans (RAPs). The core of the framework is a regulator-approved IRM strategy that is based on comprehensive pre-marketing research and serves to guide stakeholders during the post-marketing phase. The framework will assist technology developers and regulators, especially those with nascent regulatory systems, to select and implement IRM strategies that facilitate sustainable pest management.

## Introduction

Sub-Saharan Africa faces serious food security risks because its demand for cereals is expected to increase >300% by 2050 (van Ittersum et al., 2016). Maize (*Zea mays* L.) is one of the most important food crops in sub-Saharan Africa, with more than 300 million Africans depending on maize as their main food source.

One of the options for increasing maize yields is reducing losses caused by lepidopteran maize pests, such as the African maize stalk borer, *Busseola fusca* (Fuller) (Noctuidae), and the fall armyworm, *Spodoptera frugiperda* (J. E. Smith) (Noctuidae). The development and commercialization of maize hybrids that have been genetically engineered to produce *Bacillus thuringiensis* (Bt) insecticidal proteins provide a powerful tool for the control of lepidopteran maize pests. There are two key types of Bt insecticidal proteins: Cry and Vip (Chakroun et al., 2016). Cry proteins, which are produced during sporulation and form crystalline inclusions, are released when the cell wall disintegrates, whereas Vip proteins are produced and secreted during the vegetative stage of growth (Chakroun et al., 2016). Maize expressing one or more Bt insecticidal proteins is called Bt maize.

**FIGURE 1:**
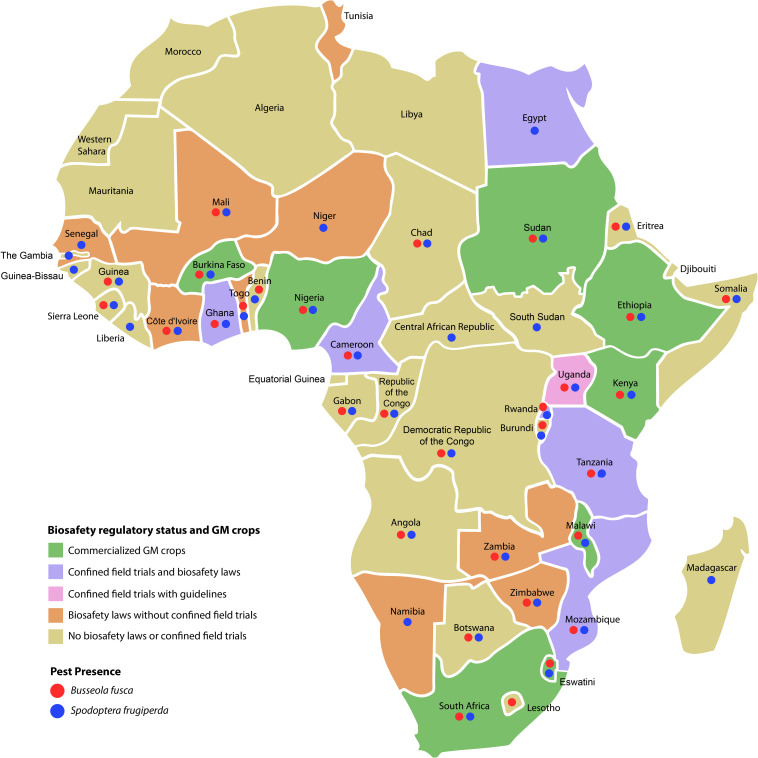
Biosafety regulatory status and GM crops in Africa. Confined field trials refer to trials involving GM crops. The presence of key lepidopteran pests of maize, *Busseola fusca* and *Spodoptera frugiperda*, in each country is shown. Data sources for construction of the figure: Biosafety regulatory status and GM crops (ABNE, 2020); Pest presence (CABI, 2020a, b).

The development, testing, and cultivation of Bt maize require functional biosafety systems, with enacted laws and adopted regimes and regulations for assessing the risks and benefits associated with genetically modified organisms (GMOs). The African Biosafety Network of Expertise (ABNE) was established to enhance the capacity of African countries to build functional biosafety regulatory systems (ABNE, 2020). However, there are significant differences in the status of the biosafety systems in different African countries (Figure 1). South Africa has a well-established GMO regulatory system, and in 1997, it became the first African country to approve commercial cultivation of Bt maize.

In South Africa, GMOs, such as Bt maize, are regulated under the GMO Act and the GMO Amendment Act (Act 23 of 2006), with the Registrar (housed within the Department of Agriculture, Land Reform and Rural Development) responsible for administering the Act. An independent, scientific Advisory Committee (AC) reviews applications and provides recommendations to the Executive Council (EC), which is the decision-making body. The opinions and perspectives in this paper are based, in part, on the author’s experiences as a member of the AC and EC, but should not be construed to be those of either the AC, EC or members of these committees.

First-generation Bt maize produces a single insecticidal protein, e.g., Cry1Ab in the case of transformation event MON810. MON810 was approved for commercial cultivation in South Africa in 1997, with resistance development in *B. fusca* noted in the 2004/2005 season. The fall armyworm developed resistance to most Bt maize hybrids just 3 years after release in Brazil (Fatoretto et al., 2017), suggesting that there is a high risk of this pest developing resistance to Bt maize also in Africa. When considering the distribution of *B. fusca* and *S. frugiperda* in Africa (Figure 1) and the fact that MON810 hybrids are being made available to African countries through the TELA Maize Project (AATF, 2020), it is highly likely that inappropriate or poorly implemented insect resistance management (IRM) programs will have significant adverse effects on the sustainable use of MON810 and other Bt maize in these African countries.

The author believes that the lessons learned from South Africa’s experience with MON810 and *B. fusca* will be of value to technology developers, regulators, and policy-makers in other countries, especially those that are developing GMO regulatory systems or have nascent systems and that are considering approving or have just approved Bt maize. This perspective paper should be seen in this context.

## The South African Experience With First-Generation Bt Maize

On the basis of studies published between 2002 and 2009, Brookes and Barfoot (2018) reported that the average yield gains in South Africa for genetically modified (GM) maize with an insect resistance (IR) trait was 11.1%. Bt maize expressing Cry1Ab was reported to provide effective control against *B. fusca* until the 2004/2005 season when severe damage to Bt maize was noted (Van Wyk et al., 2009). The reduced control (>10% damaged plants) in the 2004/2005 season was eventually attributed to the development of IR to the Cry1Ab protein in MON810 maize (Van Rensburg, 2007; Kruger et al., 2011). Although the resistance is to the Cry1Ab protein, the resistance is often simply referred to as resistance to MON810.

Based on assessments of the 2007/2008 and 2008/2009 seasons, Kruger et al. (2011) concluded that resistance to MON810 occurred in the Christiana area (North West Province) and the Vaalharts area (Northern Cape Province), areas that are approximately 50 km apart. Field-collected larvae from Vaalharts were reared, without apparent problems, for four generations on Bt maize plants (Kruger et al., 2011). By 2012, *B. fusca* populations with resistance to Cry1-Ab expressing maize occurred throughout the maize production region of South Africa (Kruger et al., 2012).

Field resistance is defined as a genetically based decrease in the susceptibility of a population to a toxin caused by field exposure to the toxin (Tabashnik, 1994). Tabashnik and Carrière (2017) classified *B. fusca* resistance in South Africa as practical resistance (field-evolved, >50% resistant individuals in a population, and reduced efficacy of Bt crop in the field). Mutations that confer resistance to Cry1Ab in Bt maize, including a dominant resistant trait, have been reported for *B. fusca* populations (Campagne et al., 2013, 2017).

Post-2015 data on resistance to MON810 in South Africa are not readily available, as by 2015 the registrant had almost completely phased out MON810 and replaced it with the pyramid event MON89034 (Cry1A.105 and Cry2Ab2).

## Factors Playing a Role in the Development of Resistance to MON810

In this section, a few factors that are likely to have played a key role in MON810-resistance development by *B. fusca* are highlighted.

Bt maize IRM programs in South Africa are almost entirely dependent on the high dose/refuge (HDR) strategy, which requires planting a refuge area composed of non-Bt maize that is in close proximity to the Bt maize field. In South Africa, there has historically been limited active engagement between applicants and regulators around IRM strategy selection. In general, applicants present generic IRM plans, developed for pests not in South Africa, rather than pest- and event-specific plans that are fully interrogated for suitability. This is problematic, as the efficacy of the HDR strategy is dependent on it being suitable for the target insect and that all the assumptions of the HDR strategy have been met (Bourguet, 2004; USEPA, 2010; Gryspeirt and Grégoire, 2012).

A crucial requirement of the HDR strategy is that the Cry protein occurs in the maize at a high concentration, preferably 25 to 50 times the LD_99_ for the target pest (Caprio et al., 2000; USEPA, 2010). Prior to commercial cultivation, to the best of the author’s knowledge, no data were available to show that the concentration of Cry1Ab in MON810 was several times the LD_99_ for South African populations of *B. fusca*.

A key principle underlying the HDR strategy is that homozygous resistant moths that may emerge from the Bt maize are more likely to mate with one of a much bigger pool of susceptible moths that emerge from the refuge area, thus producing heterozygous resistant larvae that, if inheritance is functionally recessive, are expected to be killed by the Bt maize and slow the increase of the frequency of the Bt resistance allele (Gould, 1998; Bourguet, 2004). In this context, it is important to note that South African farmers are given two options for the conventional maize refuge size: 5% (which may not be treated with an insecticide) or 20% (which may be sprayed with an insecticide or non-Bt biopesticide). In South Africa, farmers almost never choose the 20% refuge option (Kruger et al., 2009, 2012). There is insufficient empirical evidence to determine if the 5% refuge size was adequate (i.e., produced enough susceptible adults) for *B. fusca* on MON810 in South Africa. Compliance with the requirement for planting a refuge is critical for the success of the HDR strategy. In 1998, one year after commercial release of MON810, only 7.7% of farmers that planted MON810 actually planted the refuge they were legally obligated to plant (Kruger et al., 2009).

## A Framework for Development and Implementation of an Effective IRM Program

**FIGURE 2:**
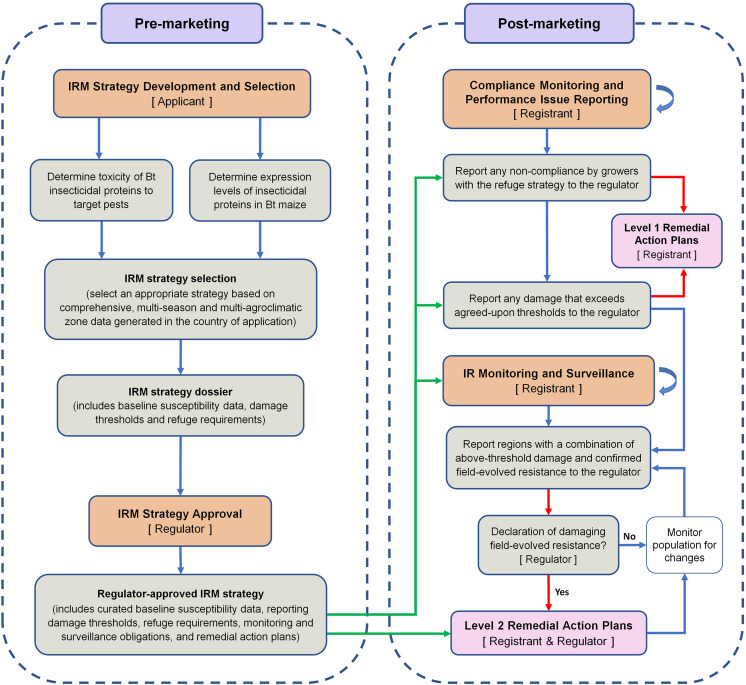
A framework for the implementation of an effective pest- and event-specific Bt maize insect resistance management program. The stakeholders responsible for key steps are shown in square brackets. The core of the framework is the regulator-approved IRM strategy dossier. Arrows: green arrows show post-marketing activities that are guided by the regulator-approved IRM strategy; red arrows show the pathways that lead to the implementation of remedial action plans; blue curved arrows show that a process continues throughout the post-marketing phase. For the sake of simplicity, it is assumed that the applicant is also the technology developer/provider. Once approval has been granted for commercial cultivation, the applicant becomes the registrant.

Based on the lessons learned from the South African experience with *B. fusca* and MON810, a framework for an effective Bt maize IRM program is proposed (Figure 2). This perspective paper does not aim to suggest a specific IRM strategy for *B. fusca* on MON810 in South Africa. Instead, the aim of the paper is to incorporate the lessons learned in a framework for developing and implementing an effective IRM program for any Bt crop–pest combination. The framework distils the overwhelming volume, especially for regulators and policymakers, of information on IRM down to a few critical steps. For readers seeking more information relating to the steps in the framework, the following references may be of use: Caprio et al. (2000), Glaser and Matten (2003), Matten et al. (2004), Head and Greenplate (2012), and Onstad (2013).

The IRM strategy development and selection phase is largely the responsibility of the applicant (usually the technology developer) that is seeking approval for commercial cultivation. There are four major parts in this selection phase (Figure 2).

The toxicity of the Bt proteins to geographically distinct populations of target pests should be determined in laboratory bioassays, using well-established bioassay methods (Siegfried et al., 2000, 2005). As Cry proteins produced by GM crops have properties that are different to naturally occurring Cry proteins or Cry proteins purified from GM bacteria (Latham et al., 2017), the choice of the Bt proteins used for these assessments needs to be carefully considered and justified by the applicant. Determination of the toxicity should include not only laboratory assessments, but assessments of the pest control provided by the Bt maize in field trials. Since the environment impacts on the expression levels of Bt proteins in Bt maize (Dutton et al., 2004; Trtikova et al., 2015), the efficacy field trials should be conducted under a range of agroclimatic conditions representative of the maize growing areas in the country. A key consideration is whether the target insects are able to complete their life cycles on the Bt maize, e.g., up to 2% of *B. fusca* larvae survived on MON810 hybrids in 1996/1997 field trials (Van Rensburg, 1999).

The expression levels of Bt proteins in Bt maize need to be determined (e.g., USEPA, 2010). To generate a complete view of the expression levels, the determinations need to be made under a range of growing conditions, ideally from the same field trials used to determine control efficacy. The expression level data generated under a range of environmental conditions will allow environment-related expression differences to be taken into consideration in the IRM strategy selection. The research should include assessment of the expression in different tissues at different plant growth stages, as expression levels in Bt maize hybrids can differ in different plant tissues at the same plant growth stage and also between the same plant tissue at different growth stages (SANBI, 2011).

The IRM strategy selection has to be based on empirical assessments of the toxicity and expression levels in the country for which commercial approval is being sought, and should take into account the population ecology of target pests (Head and Greenplate, 2012). The presence of any Cry-resistant pest populations in the country needs to be taken into consideration, as these populations may impact the efficacy of the strategy. For example, since cross-resistance among Cry1Ab and Cry1A.105 proteins is possible in lepidopteran maize pests (Hernández-Rodríguez et al., 2013), the strategy selection for MON89034 would need to consider the possibility that MON810-resistant populations are resistant to the Cry1A.105 protein in MON89034. The strategy selection cannot be based on a theoretical framework that is not supported by the in-country data. From the author’s experience, IRM is often an afterthought in the overall risk assessment dossier and IRM strategies are frequently based on generic IRM strategies and data generated in other countries for different target pests. Mathematical modeling will facilitate integration of the data and selection of a scientifically sound IRM strategy (Mallet and Porter, 1992; Gryspeirt and Grégoire, 2012; Head and Greenplate, 2012).

The IRM strategy selection and IRM strategy dossier steps are separated in the framework to highlight the importance of selecting an appropriate IRM strategy, and because the IRM strategy dossier is part of the regulatory application process rather than a research process. The applicant should prepare a comprehensive IRM strategy dossier, which lays out the IRM strategy and justifies the suitability of the strategy. The dossier should include expression data, baseline susceptibility data for representative target pest populations, refuge requirements (including refuge size and location relative to Bt maize), and damage thresholds (i.e., levels of damage that are considered unacceptable). The foundation of the dossier should be comprehensive, multi-season, and multi-agroclimatic zone data generated during confined field trials in the country of application.

In the IRM strategy approval phase, the IRM strategy dossier should be reviewed by the regulator and, in consultation with the applicant, the strategy should be refined as required. The key outcome of this phase is a regulator-approved IRM strategy. In this paper, the term regulator is used in a broad sense and may include officials from a number of government departments or agencies. In South Africa, the term regulator would refer primarily to the Office of the Registrar of the GMO Act, but also includes the EC (which consists of representatives of several government departments and the AC chairperson).

The regulator-approved IRM strategy is the core of the framework and guides the post-marketing monitoring, surveillance, and reporting. To aid in the post-marketing assessments, the approved IRM strategy should contain curated baseline susceptibility data, i.e., only baseline susceptibility data that were generated using well-established methods and are representative of the susceptibility of pest populations throughout the country of application should be included. In South Africa, baseline susceptibility data were apparently not generated, or at least not made readily available, prior to commercial cultivation of MON810. As a result, when unacceptable damage was first reported, there was disagreement as to whether the differences in control efficacy reflected natural variation in the susceptibility of *B. fusca* populations to Cry1Ab. The absence of reliable baseline susceptibility data will permanently undermine post-marketing monitoring in IRM programs.

During the review of the IRM strategy, a comprehensive remedial action plan (RAP) should be agreed upon by the applicant and the regulator. The RAPs aim to contain and, if possible, eliminate resistant populations. The Biopesticide Registration Action Documents of the US Environmental Protection Agency contain examples of RAP actions (e.g., USEPA, 2010). The IRM framework proposed in this perspective paper introduces a two-level RAP approach, level 1 (L1) and level 2 (L2), with distinctively different triggers.

There are two triggers for L1 RAPs: non-compliance by farmers with the refuge strategy or on-farm damage exceeding the agreed-upon thresholds (Figure 2). Although non-compliance and threshold-exceeding damage are reported to the regulator, L1 RAPs are immediately implemented by the registrant. In South Africa, the registrant implemented several actions in response to above-threshold damage on MON810, including: (1) heightened communication and farmer training about the importance of IRM, (2) confirming that farmers are in possession of technology and stewardship agreements and reminding farmers of their IRM obligations under these agreements, (3) increased on-farm refuge compliance monitoring and attendance of mandatory training sessions of non-compliant farmers, and (4) spraying fields with >10% damage with insecticides. These steps were taken without confirmation of field-evolved resistance development, and the registrant communicated, as early as 2007, with the regulator about the alleged resistance. These L1-type actions may be considered successful, as full refuge compliance (i.e., planting a refuge of the correct size) improved markedly after 2007 and reached 75% in the 2013/2014 season (AfricaBio presentation, 2015). In the same season, partial compliance (refuge of incorrect size planted) was 17% and non-compliance was 8%. A further indication of the effectiveness of L1 actions was that from 2010 to 2014, farmer complaints as a percentage of total hectares of MON810 planted peaked at 2.5% in the 2012/2013 season and decreased to 1.8% (≈ 49 000 ha) in the 2013/2014 season (AfricaBio presentation, 2015). During this period, the registrant and EC-initiated independent assessments kept the regulator up to date on the *B. fusca*-MON810 resistance issue.

A key part of an effective IRM program is monitoring and surveillance (Matten et al., 2004). IRM programs should ideally include pro-active monitoring, such as wide-scale application of diagnostic dose or discriminating dose assays and F_2_ screens, which are useful for the detection of rare and recessive resistance alleles (Matten et al., 2004). In the case of *B. fusca* and MON810 in South Africa, the monitoring and surveillance program had significant scope for improvement and was primarily reactive. Performance issue reporting by farmers, who are legally obligated by technology agreements to report above-threshold damage to the registrant, appeared to serve as the primary surveillance tool. The framework presented in this paper does not include above-threshold damage under the monitoring and surveillance step, but instead uses it as a trigger for L1 RAPs and the need for thorough testing of insect populations from problem sites for the presence of field-evolved resistance. In the framework, a key step under monitoring and surveillance is the reporting of regions with a combination of above-threshold damage and confirmed field-evolved resistance to the regulator, especially if the resistance is spreading rapidly. The trigger for L2 RAPs is a declaration by the regulator of damaging, field-evolved resistance. The definition of what constitutes field-evolved resistance will need to be clearly stated in the IRM strategy dossier to avoid delays in implementing L2 RAPs. For the definition step, the paper of Tabashnik et al. (2014) may be useful. L2 RAPs may include, for example, cessation of sales in the affected and bordering areas, and extensive, area-wide insecticide applications. The L2 RAPs should be proportional to the scale of the problem and should aim to safeguard the technology and prevent the spread of resistant insect populations.

In the case of *B. fusca* and MON810, populations that were suspected of having developed resistance were, to the best of the author’s knowledge, not assessed by the registrant for field-evolved resistance. However, external parties confirmed field-evolved resistance (Campagne et al., 2013). Early characterization of the resistance is important: e.g., when inheritance of resistance is non-recessive, as was the case for some *B. fusca* populations (Tabashnik and Carrière, 2017), the importance of insecticide application rather than relying on increased refuge compliance becomes apparent. Effective IRM programs should include assessments of field-evolved resistance and a clear pathway to L2 RAPs to avoid accelerated resistance evolution and rapid spread of resistance.

## Conclusion

The framework presented in this paper will facilitate the development of case-specific Bt maize IRM programs that are effective for lepidopteran maize pests. The recent arrival of *S. frugiperda* in Africa means that effective Bt maize IRM programs are crucial for African countries, as two- and three-Cry protein Bt maize pyramids lost their ability to control this pest in Brazil within 3 years after their commercial release (Fatoretto et al., 2017). By clearly defining roles for stakeholders and pathways to RAPs, the IRM framework will assist in extending the useful life of Bt maize hybrids.

## Author Contributions

The author confirms being the sole contributor of this work and has approved it for publication.

## Conflict of Interest

The author declares that the research was conducted in the absence of any commercial or financial relationships that could be construed as a potential conflict of interest.

## Abbreviations

AC: Advisory Committee
EC: Executive Council
GM: genetically modified
GMO: genetically modified organism
HDR: high dose/refuge
IR: insect resistance
IRM: insect resistance management
RAPs: remedial action plans.
